# Performance of a broth microdilution assay for routine minimum inhibitory concentration determination of 14 anti-tuberculous drugs against the *Mycobacterium tuberculosis* complex based on the EUCAST reference protocol

**DOI:** 10.1128/aac.00946-24

**Published:** 2024-12-18

**Authors:** Mikael Mansjö, Carmen Espinosa-Gongora, Ishak Samanci, Ramona Groenheit, Jim Werngren

**Affiliations:** 1 Public Health Agency of Sweden 25545 , Solna, Sweden; 2 ECDC Fellowship Programme, Public Health Microbiology path (EUPHEM), European Centre for Disease Prevention and Control (ECDC) 56756 , Stockholm, Sweden; St. George's, University of London, London, United Kingdom

**Keywords:** tuberculosis, broth microdilution, MIC determination, antimicrobial susceptibility

## Abstract

This comparative study aimed at qualifying a broth microdilution (BMD) assay for phenotypic drug susceptibility testing (pDST) of *Mycobacterium tuberculosis* complex (MTBC) strains for implementation in a routine DST workflow. The assay was developed based on the EUCAST (European Committee on Antimicrobial Susceptibility Testing) reference protocol for determination of the minimum inhibitory concentration (MIC) of 14 anti-tuberculous drugs (isoniazid [INH], rifampicin [RIF], ethambutol [EMB], amikacin [AMI], moxifloxacin [MFX], levofloxacin [LFX], bedaquiline [BDQ], clofazimine [CFZ], delamanid [DLM], pretomanid [PA], para-aminosalicylic acid [PAS], linezolid [LZD], ethionamide [ETH], and cycloserine [CS]). Forty MTBC strains with various drug resistance profiles were tested to determine the agreement between MIC results and genotypic drug susceptibility testing (gDST) results derived from whole-genome sequencing (WGS). The agreement between the BMD and gDST results was solid for the majority of the drugs (average agreement 98%, range 90%–100%), including key drugs such as INH, RIF, MFX, LFX, BDQ, DLM, and PA. Ten discrepancies were identified (corresponding to 1.8% of the total number of MIC determinations) and most (8/10) were characterized by MICs equal or close to the potential critical concentration (pCC) applied in the BMD assay. Importantly, the assay can be adjusted to new drug recommendations and concentrations, tailored to local needs. We conclude that the BMD assay provides reliable results, and its implementation in our MTBC routine workflow will produce valuable data that improve our understanding and management of MTBC drug resistance.

## INTRODUCTION

At present, phenotypic drug susceptibility testing (pDST) of the *Mycobacterium tuberculosis* complex (MTBC) is, in most countries, heavily dependent on the BACTEC MGIT 960 system. Although MGIT remains a valuable solution in settings and laboratories that are dependent on an automated system for pDST, this method is subject to limitations such as the inherent weaknesses of binary pDST methods (e.g., detection of low-level resistance) (1–3). Furthermore, recent studies piloting broth microdilution (BMD) assays for pDST of MTBC predominantly used commercial standardized or customized BMD plates (1, 4–6), offering limited flexibility for adjustments to suit individual laboratory or epidemiological needs and creating dependence on the availability of specific commercial products.

In the past, critical concentrations (CCs) used for susceptibility testing of MTBC have been experimentally determined on microbiological and clinical evidence but only for certain drugs and culture media with a methodological testing variation that has complicated the use of these values (7–9). Due to the lack of a reference method for determination of the minimum inhibitory concentration (MIC) of MTBC historically, the EUCAST-AMST (European Committee on Antimicrobial Susceptibility Testing – subcommittee on Antimycobacterial Susceptibility Testing) developed a protocol for BMD testing that was endorsed by EUCAST as the reference method to set quality control ranges, epidemiological cutoffs (ECOFFs), and clinical breakpoints (CBs) for the MTBC (10). MIC determination is used to assess the ECOFF, defined as the highest MIC value measured for phenotypically wild-type isolates, i.e., those without acquired resistance mechanisms (11). EUCAST sets CBs by evaluating ECOFFs, pharmacokinetic/pharmacodynamic (PK/PD) and clinical outcome data together (12). In parallel, there have been other initiatives aiming at determining ECOFFs for MTBC BMD assays (13–16). The CCs used for DST of MTBC are in turn defined by the World Health Organization (WHO) who review and quality assure MIC and ECOFF data sets reported from laboratories using different methods (17). The EUCAST reference method for determining the MIC of MTBC was established in 2019 and to date, sufficient data are not available to endorse CCs for this method.

During the last decade, the development and implementation of robust pipelines for genotypic drug susceptibility testing (gDST) of MTBC based on whole-genome sequencing (WGS) data have prompted significant changes in diagnostic algorithms (18, 19). These pipelines have proven their ability to reliably predict the phenotypic drug resistance pattern of MTBC isolates (1, 20, 21), and settings such as the Netherlands and New York State have already adopted diagnostic algorithms where pDST has been substantially reduced (22, 23). In Sweden, a typical low-incidence setting with 3.4 cases of tuberculosis (TB) per 100,000 inhabitants as reported in 2023 (24), a similar adjustment of the diagnostic algorithm is currently underway. Essentially, in order to fully optimize the potential of gDST, MTBC isolates will be sequenced locally at clinical TB laboratories, and those with at least one known or ambiguous resistance mutation (approximately 15% of MTBC isolates per year [23]) will be forwarded to the National Reference Laboratory for tuberculosis at the Public Health Agency of Sweden for extended pDST.

Here, we present a comparative study where a BMD assay, based on the EUCAST reference protocol (10) and used for determining the MIC of MTBC, was assessed against our routine gDST. The assay’s agreement with gDST was evaluated using a panel of 40 MTBC strains covering various resistance profiles, with the objective of qualifying the assay for implementation in our routine workflow.

## MATERIALS AND METHODS

### Selection of MTBC isolates

Thirty-four clinical strains, five quality-control (QC) strains, and one vaccine strain (BCG SSI 1331) from the MTBC strain collection at the Public Health Agency of Sweden were included in the study. The test panel encompassed MTBC lineages 1 (*n* = 9), 2 (*n* = 8), 3 (*n* = 4), 4 (*n* = 13), 5 (*Mycobacterium africanum*, *n* = 1), and 7 (*n* = 1) in addition to two mixed genotypes (lineage 2 + 4 and lineage 1 + 4) and two *Mycobacterium bovis* (both BCG) strains (25). The collection comprises a mix of drug-susceptible and drug-resistant strains (21 susceptible to all drugs, 12 multidrug resistant [MDR], 1 extensively drug resistant [XDR], and 6 non-MDR displaying resistance to one or more drugs) as determined by routine testing with BACTEC MGIT 960, Löwenstein Jensen proportion method, or BACTEC 460. The lineage classification and the results from the routine pDST are presented in the supplementary material (Table S1).

### Genotypic drug susceptibility testing

DNA extraction and WGS were performed as previously described (26), except raw Illumina reads from strains SEA200400001 and BCG SSI 1331, which were obtained from the Sequence Read Archive and the European Nucleotide Archive, respectively. Accession numbers are available in Table S1.

The sequencing data were screened for mutations present in our in-house mutation catalog. The catalog is divided into one set of well-characterized resistance mutations (Table S2) (isolates with such a mutation are classified as genotypically resistant to the corresponding drug/s) and one set of ambiguous or less well-known mutations (Table S3) that might play a role in phenotypic resistance (described as candidate mutations in this article). The mutation catalog was first assembled prior to the release of the first edition of the WHO mutation catalog (27) but has since been updated according to the WHO documents (essentially, all mutations in groups 1 and 2 not already present in the in-house catalog have been added to either the list of well-characterized mutations or the candidate list). In detail, raw sequencing reads were mapped against genes and regions from the H37Rv NC_000962.3 reference genome covering the mutations present in the mutation catalog (CLC Assembly Cell version 4.4.2, QIAGEN, Aarhus, Denmark). The extracted variants (CLC Assembly Cell version 4.4.2, QIAGEN, Aarhus, Denmark) were filtered (in-house script; minimum frequency of reads calling single nucleotide polymorphisms: 10%; minimum frequency of reads calling insertions and deletions: 80%), and the remaining variants were compared against the in-house mutation catalog.

### Broth microdilution assay

**Fig 1:**
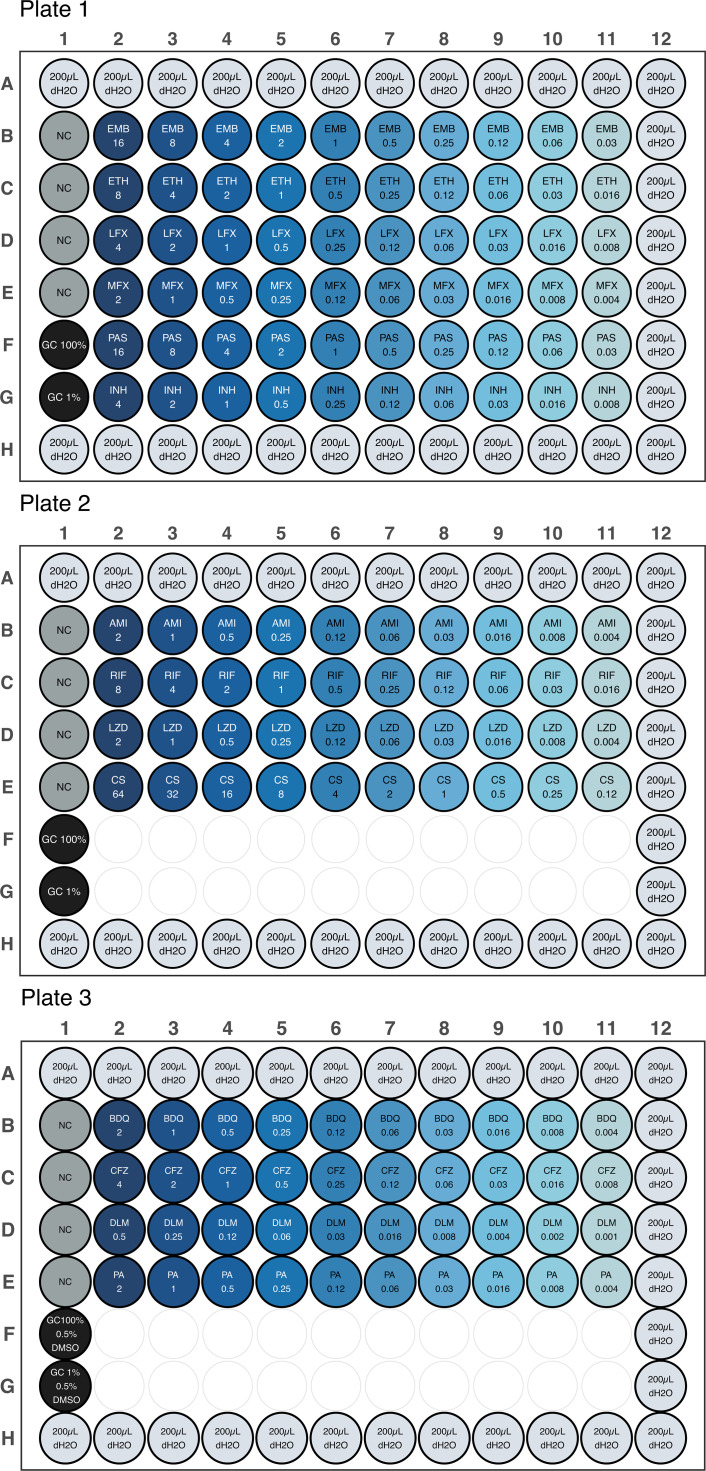
Layout of the broth microdilution (BMD) assay for the determination of minimum inhibitory concentration of 14 anti-tuberculous drugs. The assay is distributed in three 96-well plates. Plates 1 and 2 include water-soluble drugs isoniazid (INH), rifampicin (RIF), ethambutol (EMB), amikacin (AMI), moxifloxacin (MFX), levofloxacin (LFX), para-aminosalicylic acid (PAS), linezolid (LZD), ethionamide (ETH), and cycloserine (CS). Plate 3 includes non-water-soluble drugs bedaquiline (BDQ), clofazimine (CFZ), delamanid (DLM), and pretomanid (PA). The concentration of each drug is displayed in its corresponding well in milligrams per liter (mg/L). All plates include negative controls (NC) and growth controls (GC1% and GC100%). Plate 3 also contained 0.5% dimethyl sulfoxide (DMSO).

A detailed protocol for the BMD assay is provided in the Supplementary methods, and the preparation of drug stock solutions is presented in Table S4. The final assay layout is shown in Figure 1.

In summary, the assay included two plates for water-soluble drugs isoniazid (INH), rifampicin (RIF), ethambutol (EMB), amikacin (AMI), moxifloxacin (MFX), levofloxacin (LFX), para-aminosalicylic acid (PAS), linezolid (LZD), ethionamide (ETH), and cycloserine (CS), along with one plate for non-water-soluble drugs bedaquiline (BDQ), clofazimine (CFZ), delamanid (DLM), and pretomanid (PA). MIC testing was performed using Middlebrook 7H9 supplemented with 10% oleic acid dextrose catalase (OADC) and 0.2% glycerol. All drugs were tested at 10 concentrations, and bacterial inoculums were prepared according to the EUCAST reference protocol (10). Four negative controls and two growth controls (GC1% and GC100%) were included in each BMD plate.

The plates were incubated at 36°C ± 1°C and read at day 7, 10, 14, and 21. The MICs were determined at the point the GC1% well showed visible growth.

The reference strain H37Rv ATCC 27294 was tested on eight occasions to examine the technical reproducibility of the assay (including the determination of colony forming unit [CFU] counts for each replicate).

### Data analysis

Results of the BMD assay were used to classify MTBC strains as susceptible or resistant using the potential critical concentrations (pCCs) specified in Table S5, including references. Because established CCs are lacking for the BMD assay described here, the pCCs were derived from other BMD studies and the recommended test concentrations in MGIT. Importantly, these pCCs have not been endorsed by any breakpoint committee and are therefore highly preliminary and used for comparison in the present study only.

MTBC strains carrying at least one well-characterized resistance mutation present in the mutation catalog were considered resistant to a specific drug. The association between each candidate resistance mutation and BMD results were discussed separately. All calculations and plots were performed using the epiR and ggplot2 packages in R version 4.2.2 (28–30).

## RESULTS

The agreement between the BMD assay (based on pCC) and gDST for all 14 anti-TB drugs ranged from 90% to 100% with an average of 98%, as shown in Table 1. All drugs but LZD had at least one strain displaying genotypic or phenotypic resistance, and strains carrying known resistance mutations displayed consistent phenotypic resistance for eight drugs (INH, RIF, AMI, MFX, LFX, DLM, PA, and PAS). A list of discrepant genotypic and phenotypic results is given in Table 2. Among the 10 identified discrepancies (corresponding to 1.8% of the total number of MIC determinations), eight strains (seven of them judged as genotypically resistant) had an MIC equal or close to the pCC, and four of them involved EMB. The two strains (XTB_16-019 and XTB_16-001) that obtained a mixed genotype in the lineage classification displayed resistance mutations in a fraction of the sequencing reads (frequency ranging from 21% to 86%) (Table S1). Additionally, low sequencing depth did not allow a conclusive determination of the *ethA* genotype in one strain (XTB_16-019).

**TABLE 1:** Two-by-two tables showing the classification of 40 *Mycobacterium tuberculosis* complex strains as genotypically resistant or susceptible (gR or gS) based on whole-genome sequencing, and phenotypically resistant or susceptible (pR or pS) using the broth microdilution assay containing 14 anti-tuberculous drugs^*a*^

Drug^*b*^		gR	gS	Agreement
AMI	pR	1	0	100%
	pS	0	39	
BDQ	pR	3	0	97%
	pS	1	35	
CFZ	pR	2	0	97%
	pS	1	36	
CS	pR	0	0	95%
	pS	2	37	
DLM	pR	5	0	100%
	pS	0	34	
EMB	pR	6	0	90%
	pS	4	29	
ETH	pR	7	0	97%
	pS	1	27	
INH	pR	14	0	100%
	pS	0	26	
LFX	pR	2	0	100%
	pS	0	38	
LZD	pR	0	0	100%
	pS	0	40	
MFX	pR	2	0	100%
	pS	0	38	
PA	pR	5	0	100%
	pS	0	34	
PAS	pR	2	1	97%
	pS	0	36	
RIF	pR	15	0	100%
	pS	0	25	

^*a*^ The percentage agreement between the two methods is included. Phenotypic classification was determined using the potential critical concentrations (pCC) listed in Table S5. Strains carrying candidate mutations are excluded.

^*b*^ AMI, amikacin; BDQ, bedaquiline; CFZ, clofazimine; CS, cycloserine; DLM, delamanid; EMB, ethambutol; ETH, ethionamide; INH, isoniazid; LFX, levofloxacin; LZD, linezolid; MFX, moxifloxacin; PA, pretomanid; PAS, para-aminosalicylic acid; and RIF, rifampicin.

**TABLE 2:** List of discrepancies observed between genotypic drug susceptibility testing (gDST), based on identification of resistance mutations by whole-genome sequencing, and minimum inhibitory concentrations (MICs) determined by our broth microdilution (BMD) assay^*a*^

Drug^*b*^	Sample ID	gDST	BMD MIC (mg/L)	Possible explanation(s) behind discrepancy
EMB	SEA201600522	*embB*: Met306Ile	4	MIC close to the potential critical concentration; *embB* resistance mutations can confer MICs around 4 mg/L (4, 13)
	SEA201700328	*embB*: Met306Val	4	MIC close to the potential critical concentration; *embB* resistance mutations can confer MICs around 4 mg/L (4, 13)
	XTB_16-019	*embB*: Asp354Ala (77%)	4	Mixed genotype; DNA not extracted from the same subculture as the BMD inoculum was prepared from (higher proportion of the susceptible population in the BMD assay?); MIC close to the potential critical concentration. *embB* resistance mutations can confer MICs around 4 mg/L (4, 13)
	XTB_16-020	*embB*: Asp354Ala	4	MIC close to the potential critical concentration; *embB* resistance mutations can confer MICs around 4 mg/L (4, 13)
BDQ	XTB_16-019	*atpE*: Glu61Asp (21%)	0.016	Mixed genotype; DNA not extracted from the same subculture as the BMD inoculum was prepared from (higher proportion of the susceptible population in the BMD assay?)
CFZ	XTB_16-001	*Rv0678*: Leu136Pro (50%)	1.0	MIC close to the potential critical concentration; mixed genotype; DNA not extracted from the same subculture as the BMD inoculum was prepared from (higher proportion of the susceptible population in the BMD assay?)
PAS	*M. bovis* BCG SSI 1331	No resistance mutation or candidate mutation identified	2	MIC close to the potential critical concentration; trailing endpoints for PAS make MIC interpretation difficult in this case
ETH	SEA201800080	*ethA*: Met1Arg	0.5	Mutation also reported in many ETH susceptible isolates (27)
CS	SEA200400001 (BCG)	*cycA*: Gly122Ser	32	MIC close to the potential critical concentration; BCG strains typically have a reduced susceptibility for CS (31, 32), but the MIC distribution in BMD assays has not been thoroughly studied
	*M. bovis* BCG SSI 1331	*cycA*: Gly122Ser	16	MIC close to the potential critical concentration; BCG strains have a reduced susceptibility for CS (31, 32), but the MIC distribution in BMD assays has not been thoroughly studied

^*a*^ The table includes possible explanations for the discrepancies as judged by the authors.

^*b*^ EMB, ethambutol; BDQ, bedaquiline; CFZ, clofazimine; PAS, para-aminosalicylic acid; ETH, ethionamide; and CS, cycloserine.

**Fig 2:**
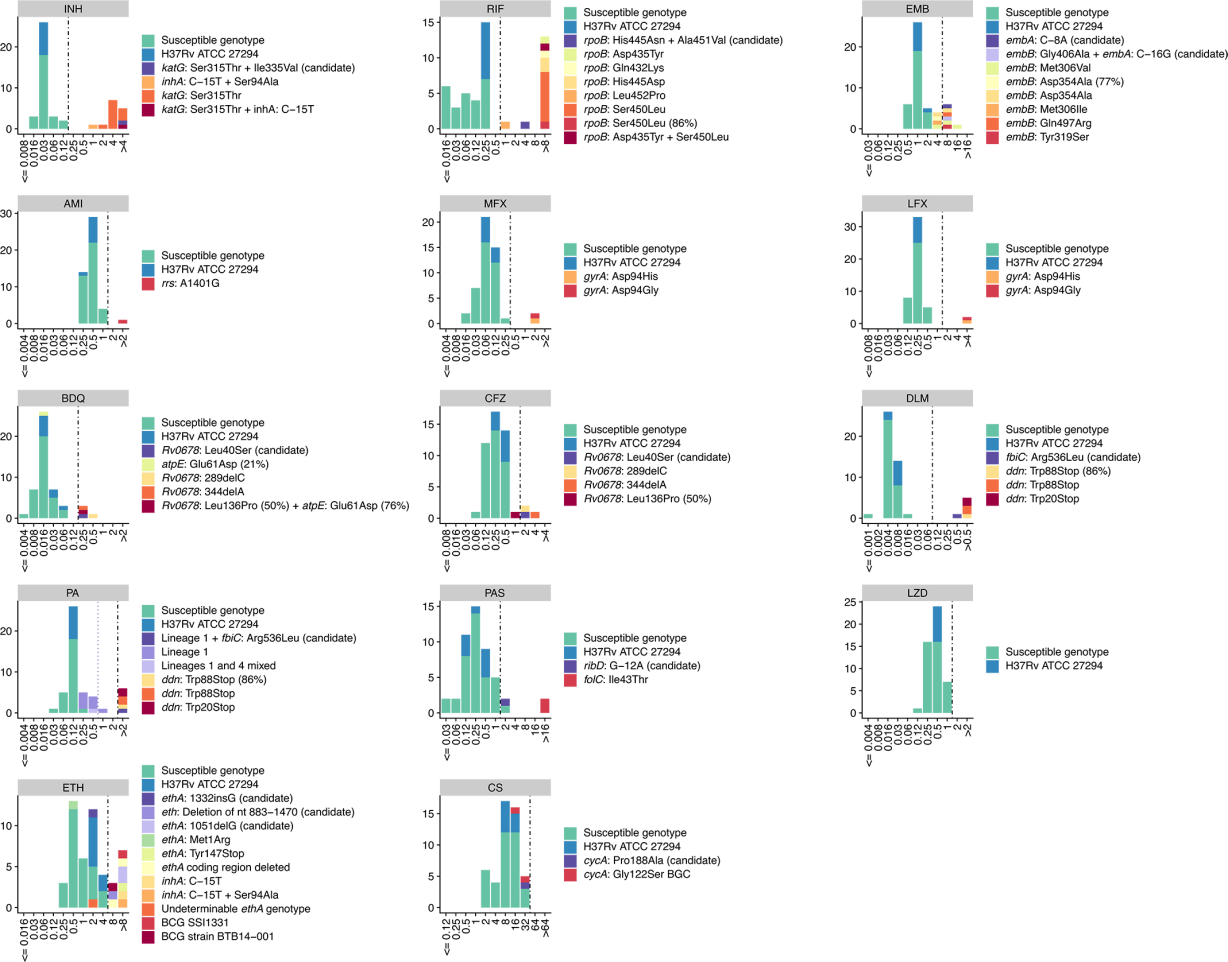
Minimum inhibitory concentration (MIC) of a panel of 40 *Mycobacterium tuberculosis* complex (MTBC) strains against 14 anti-tuberculous drugs in the presence/absence of resistance mutations as determined by whole-genome sequencing, as determined by the broth microdilution (BMD) assay developed based on the EUCAST reference method. Susceptible genotypes and presence of specific resistance mutations or MTBC lineages are indicated by colors for each drug. Eight technical replicates of reference strain H37Rv ATCC27294 are included alongside the panel of 40 strains. The 14 drugs included isoniazid (INH), rifampicin (RIF), ethambutol (EMB), amikacin (AMI), moxifloxacin (MFX), levofloxacin (LFX), bedaquiline (BDQ), clofazimine (CFZ), delamanid (DLM), pretomanid (PA), para-aminosalicylic acid (PAS), linezolid (LZD), ethionamide (ETH), and cycloserine (CS). Drug concentrations are given in milligrams per liter. Potential critical concentrations (pCCs) are indicated by black vertical dot-dash lines. For PA, the purple vertical dotted line indicates an alternative MGIT test concentration (0.5 mg/L) according to the WHO policy statement (33).

MIC distributions produced by the BMD assay are shown in Figure 2, including the presence of known and candidate resistance mutations. Importantly, all strains classified as fully susceptible by gDST were also susceptible to all drugs in the BMD assay, and all MDR/RIF-monoresistant strains were correctly identified as such by the BMD assay. Resistance phenotypes of strains carrying candidate mutations are shown in Table 3, including the BMD assay and prior MGIT or Löwenstein Jensen proportion results. Notably, the BMD assay had to be repeated for the two strains carrying the *folC* mutation Ile43Thr (associated with PAS resistance) because of the slow growth (the impaired growth rate was also observed in the routine MGIT testing). All resistance mutations identified in our MTBC panel and their corresponding categorical pDST and BMD results are listed in Table S1.

**TABLE 3:** Resistance phenotype of strains carrying candidate mutations (S = susceptible, R = resistant), including the broth microdilution assay (BMD) and prior relevant phenotypic drug susceptibility tests, BACTEC MGIT 960 (MGIT) or Löwenstein Jensen proportion method (LJ prop)^*a*^

Drug^*b*^	Genotype	MGIT	MGIT CC	BMD	BMD MIC	BMD pCC
EMB	*embA*: C-8A	S	5	R	8	4
BDQ	*Rv0678*: Leu40Ser	R	1	R	0.25	0.12
CFZ	*Rv0678*: Leu40Ser	R	1	R	2	1
DLM	*fbiC*: Arg536Leu	R	0.06	R	0.5	0.06
PAS	*ribD*: G-12A	R	4^*c*^	R	2	1
ETH	*ethA*: Deletion of nt 883-1470	S	5	R	8	4
ETH	*ethA*: 1332insG	S	5	S	2	4
ETH	*ethA*: 1051delG	S	5	R	>8	4
ETH	*ethA*: 1051delG	S	5	R	>8	4
PA	Lineage 1 + *fbiC*: Arg536Leu	Not available	0.5 and 2^*d*^	R	>2	2

Drug^*b*^	Genotype	LJ prop	LJ prop CC	BMD	BMD MIC	BMD pCC
CS	*cycA*: Pro188Ala	S	30^*e*^	S	32	32

^*a*^ Potential critical concentration (pCC) used and minimum inhibitory concentration (MIC) are provided in milligrams per liter. Each row represents a strain. Strains carrying candidate mutations in combination with known resistance mutations within each specific drug are excluded from the table.

^*b*^ EMB, ethambutol; BDQ, bedaquiline; CFZ, clofazimine; DLM, delamanid; PAS, para-aminosalicylic acid; ETH, ethionamide; PA, pretomanid; and CS, cycloserine.

^*c*^ Provisional CC.

^*d*^ Recommended test concentrations according to the World Health Organization (WHO) policy statement.

^*e*^ CC not endorsed by the WHO.

A broad assessment of the agreement between routine pDST and gDST was performed for a subset of drugs with sufficient MGIT data, showing a strong agreement for INH (100%, *n* = 40), RIF (95%, *n* = 40), and PA (100%, *n* = 31). Agreement was slightly lower for EMB (89%, *n* = 37), in line with previously reported poor categorical agreement between pDST and gDST for this drug (34) (Table S1).

For 5 out of 14 anti-tuberculous drugs, the MICs of the eight H37Rv ATCC27294 replicates displayed the same MIC. Among the remaining drugs, the H37Rv replicates exhibited either two consecutive MIC (seven drugs) or three consecutive MIC (two drugs). In all instances, H37Rv replicates fell within the susceptible category (Fig. 2). The CFU counts were also within the acceptable range specified in the EUCAST reference protocol for seven out of eight H37Rv replicates (100–140 CFUs from plating 10 microliters of the 10^−3^ dilution of the McFarland 0.5 inoculum suspension) (10). The remaining replicate was non-determinable due to a growth problem on the 7H10 agar.

## DISCUSSION

Adequate CCs are a crucial aspect of pDST, and generating abundant BMD reference MIC data is essential to improve accuracy and relevance, and to develop guidelines that enhance the utility and standardization of MTBC pDST across laboratories. In addition to supporting the implementation of adequate CCs, our workflow will provide a combination of genotypic and phenotypic data that will improve our understanding of the association between specific mutations and MIC values. This knowledge will help us optimize the value of gDST and facilitate timely treatment decisions. Moreover, such information will become available for isolates with novel or candidate mutations, contributing to resolving or identifying gaps in mutation catalogs.

The BMD assay was able to assign MIC data in agreement with the presence/absence of known resistance mutations for a majority of the strains and drugs tested. Most discrepancies between BMD and gDST results (9/10) were susceptible phenotypes in strains with known resistance mutations. Among the discrepancies, eight strains displayed MICs adjacent to the critical concentration of the corresponding drug, highlighting the importance of non-binary susceptibility data and shedding light on the impact of specific mutations on susceptibility, such as some of the observed *embB* mutations, which confer MICs very close to the EMB pCC. In the Swedish diagnostic algorithm, these gDST results would overrule the pDST results, and the drug in question should consequently be excluded from the treatment regimen. The discrepancies observed in the strains with resistance mutations at frequencies <80% would benefit from a combined workflow with genotypic and phenotypic methods as described above. Another discrepancy consisting of an ETH MIC three twofold dilutions below the pCC (0.5 mg/L) while carrying the *ethA* mutation Met1Arg further supported the notion that this mutation confers variable ETH phenotypes (27). Furthermore, regarding ETH, we observed that both BCG strains in the test panel exhibited MICs above the pCC, a finding in line with a previous study where both BCG SSI 1331 and BCG Connaught were resistant to ETH at 5 mg/L in BACTEC MGIT 960 (35).

To our knowledge, this study is the first to provide PAS susceptibility testing data using the EUCAST reference method. Previous studies showed poor reproducibility of PAS pDST by other BMD methods (5), partly because of the difficulty in interpreting trailing endpoints due to the bacteriostatic effect of this drug (36), and the WHO has advised against its inclusion in BMD assays (37). Our PAS BMD results showed an MIC distribution with a notable gap between susceptible genotypes and strains harboring known resistance mutations (Fig. 2). Two strains (one susceptible genotype and one strain carrying a candidate mutation) presented an MIC of 2 mg/L, placing them next to the susceptible population, yet one dilution step above the pCC of 1 mg/L. Susceptible genotypes with PAS MICs above the pCCs have also been observed by others (4). Adjusting the pCC to 2 mg/L would fully corroborate the genotypic results in our study, but our strain collection had only two strains with known PAS resistance mutations, which does not allow us to confirm the MIC gap or propose a pCC of 2 mg/L (other resistance mutations could arguably confer MIC values around 2–4 mg/L). Additionally, the presence of mutations not yet cataloged for PAS cannot be ruled out. Of relevance, MIC determination of our strains with *folC* Ile43Thr mutation required the use of completely fresh bacterial inocula due to the slow growth seen in this genotype (*folC* has previously been predicted to be essential for *in vitro* growth of *M. tuberculosis* [38]).

Second, the EMB MIC of 4 mg/L observed in four strains carrying *embB* mutations supports previous suggestions that the EMB pCC of 4 mg/L may be too high (4) and the proposal for introducing a borderline category (13). Alternatively, an area of technical uncertainty (ATU) according to the EUCAST guidelines (39) could be discussed for this MIC, in line with the “inconclusive” category introduced for the commercially available MIC plates by the Clinical and Laboratory Standards Institute (CLSI) (40). Further studies are, however, needed to understand which *embB* mutations confer MICs below 8 mg/L, and whether EMB can be used for treatment in these instances. Third, historic pDST using MGIT and other BMD methods has described elevated PA MICs in MTBC lineage 1 strains lacking resistance mutations (41, 42). Similar to other studies (42), the BMD assay used here assigned elevated PA MIC values to lineage 1 strains, but 9/10 strains still remained in the susceptible category (PA MIC ≤2). The remaining lineage 1 strain was phenotypically resistant (PA MIC >2 mg/L) with the *fbiC* mutation Arg536Leu. The clinical relevance of the elevated PA MIC values in lineage 1 is still under consideration, with no definitive decision made yet by the WHO (42). Fourth, both strains harboring the so-called borderline *rpoB* mutations (His445Asn together with Ala451Val in SEA201600522, and Leu452Pro in SEA23-09441) had RIF MICs well above the pCC. This contrasts the susceptible phenotypes obtained in the routine MGIT test, possibly indicating a higher sensitivity of the BMD assay to detect RIF resistance in strains with borderline *rpoB* mutations, but this finding remains to be confirmed by other studies (34). Last, the MIC distribution for several drugs (e.g., AMI, PA, LFX, LZD, and ETH) suggests further improvement of the assay layout by adjusting the drug concentration ranges upward in order to avoid truncation of the MIC values for resistant strains and thereby provide a more comprehensive view of the susceptibility profiles and improve treatment decision-making accuracy.

We were able to investigate the effect of candidate mutations on MIC in 10 out of 13 mutations (Table 3), excluding those presenting in combination with other mutations that confer resistance to a specific drug. A majority of these candidate mutations (8/10) conferred MICs above the pCC. The same pattern extends to the PA candidate *fbiC* mutation Arg536Leu, although this mutation was present within a lineage 1 strain, potentially correlating with a higher MIC.

The main study limitation was our sample size, which was relatively small and included a low number of resistant isolates for several drugs. Moreover, the lack of established CCs for BMD assays obstructs a thorough performance analysis. For PA, we adopted the recommended test concentrations in MGIT (33), and the agreement between BMD and gDST was determined by using a pCC of 2 mg/L, partly to overcome the issue of elevated PA MICs for lineage 1 strains. It is clear that more robust recommendations regarding CCs for BMD assays are urgently needed, and future studies can hopefully contribute to confirm the validity of the pCCs used here or else refine them (notably, the EUCAST guidelines for setting for ECOFF values state that the MIC distributions used for setting an ECOFF value must be based on the MIC data from at least five laboratories [43]). Nevertheless, we consider the assay sufficiently robust for integration into our routine DST algorithm.

In conclusion, the BMD assay shows the feasibility and reliability to perform successfully in combination with gDST as part of our MTBC susceptibility testing workflow, generating valuable data to enhance the understanding and management of MTBC drug resistance. Furthermore, MIC data generated with the EUCAST reference protocol are scarce, and the present study will, together with its implementation in our routine workflow, contribute to the body of knowledge regarding MIC distributions for clinical MTBC isolates. Although commercial plates for pDST offer a higher degree of standardization, the BMD assay presented in this study provides specialized TB laboratories with the flexibility to promptly adjust to new drug recommendations and concentration ranges, tailored to local needs. We argue that the adaptability, regarding concentration ranges and set of included drugs, of this BMD assay outweighs the advantages of the currently available commercial assay for MIC determination of MTBC. Preferably, isolates should be prioritized based on gDST, using methods such as WGS or screening by PCR, and selected for extended MIC determination if the identified mutation/s has implications for the choice of treatment regimen (the BMD assay may be less clinically relevant for isolates that are genotypically susceptible to all first-line drugs, even when gDST indicates resistance to second-line drugs like DLM and PA, although it remains valuable for tracking the emergence of resistance and monitoring trends).

## SUPPLEMENTAL MATERIAL

Supplemental methods aac.00946-24-s0001.docx Detailed description of the BMD protocol.

Table S1 aac.00946-24-s0002.xlsx Results from routine phenotypic and genotypic tests, and accessions.

Table S2 aac.00946-24-s0003.xlsx In-house mutation catalog used for the genotypic DST.

Table S3 aac.00946-24-s0004.xlsx List of candidate mutations.

Table S4 aac.00946-24-s0005.xlsx Dilution of antibiotic stock and working solutions.

Table S5 aac.00946-24-s0006.xlsx The potential critical concentrations used in the BMD assay.

ASM does not own the copyrights to Supplemental Material that may be linked to, or accessed through, an article. The authors have granted ASM a non-exclusive, world-wide license to publish the Supplemental Material files. Please contact the corresponding author directly for reuse.

## References

[1] Finci I, Albertini A, Merker M, Andres S, Bablishvili N, Barilar I, Cáceres T, Crudu V, Gotuzzo E, Hapeela N, et al.. 2022. Investigating resistance in clinical Mycobacterium tuberculosis complex isolates with genomic and phenotypic antimicrobial susceptibility testing: a multicentre observational study. Lancet Microbe 3:e672–e682. doi:10.1016/S2666-5247(22)00116-1 35907429 PMC9436784

[2] Grobbel HP, Merker M, Köhler N, Andres S, Hoffmann H, Heyckendorf J, Reimann M, Barilar I, Dreyer V, Hillemann D, Kalsdorf B, Kohl TA, Sanchez Carballo P, Schaub D, Todt K, Utpatel C, Maurer FP, Lange C, Niemann S. 2021. Design of multidrug-resistant tuberculosis treatment regimens based on DNA sequencing. Clin Infect Dis 73:1194–1202. doi:10.1093/cid/ciab359 33900387 PMC8492214

[3] Heyckendorf J, Andres S, Köser CU, Olaru ID, Schön T, Sturegård E, Beckert P, Schleusener V, Kohl TA, Hillemann D, Moradigaravand D, Parkhill J, Peacock SJ, Niemann S, Lange C, Merker M. 2018. What is resistance? Impact of phenotypic versus molecular drug resistance testing on therapy for multi- and extensively drug-resistant tuberculosis. Antimicrob Agents Chemother 62:e01550-17. doi:10.1128/AAC.01550-17 PMC5786814 29133554

[4] Nonghanphithak D, Kaewprasert O, Chaiyachat P, Reechaipichitkul W, Chaiprasert A, Faksri K. 2020. Whole-genome sequence analysis and comparisons between drug-resistance mutations and minimum inhibitory concentrations of Mycobacterium tuberculosis isolates causing M/XDR-TB. PLoS One 15:e0244829. doi:10.1371/journal.pone.0244829 33382836 PMC7775048

[5] Rancoita PMV, Cugnata F, Gibertoni Cruz AL, Borroni E, Hoosdally SJ, Walker TM, Grazian C, Davies TJ, Peto TEA, Crook DW, Fowler PW, Cirillo DM, CRyPTIC Consortiumfor the CRyPTIC Consortiumfor the CRyPTIC Consortium. 2018. Validating a 14-drug microtiter plate containing bedaquiline and delamanid for large-scale research susceptibility testing of Mycobacterium tuberculosis. Antimicrob Agents Chemother 62:e00344-18. doi:10.1128/AAC.00344-18 29941636 PMC6125532

[6] Davies Forsman L, Niward K, Hu Y, Zheng R, Zheng X, Ke R, Cai W, Hong C, Li Y, Gao Y, Werngren J, Paues J, Kuhlin J, Simonsson USH, Eliasson E, Alffenaar JW, Mansjö M, Hoffner S, Xu B, Schön T, Bruchfeld J. 2018. Plasma concentrations of second-line antituberculosis drugs in relation to minimum inhibitory concentrations in multidrug-resistant tuberculosis patients in China: a study protocol of a prospective observational cohort study. BMJ Open 8:e023899. doi:10.1136/bmjopen-2018-023899 PMC6173237 30287613

[7] Schön T, Miotto P, Köser CU, Viveiros M, Böttger E, Cambau E. 2017. Mycobacterium tuberculosis drug-resistance testing: challenges, recent developments and perspectives. Clin Microbiol Infect 23:154–160. doi:10.1016/j.cmi.2016.10.022 27810467

[8] Schön T, Köser CU, Werngren J, Viveiros M, Georghiou S, Kahlmeter G, Giske C, Maurer F, Lina G, Turnidge J, van Ingen J, Jankovic M, Goletti D, Cirillo DM, Santin M, Cambau E, ESGMYC. 2020. What is the role of the EUCAST reference method for MIC testing of the Mycobacterium tuberculosis complex? Clin Microbiol Infect 26:1453–1455. doi:10.1016/j.cmi.2020.07.037 32768492

[9] Canetti G, Froman S, Grosset J, Hauduroy P, Langerova M, Mahler HT, Meissner G, Mitchison DA, Sula L. 1963. Mycobacteria: laboratory methods for testing drug sensitivity and resistance. Bull World Health Organ 29:565–578. 14102034 PMC2555065

[10] Schön T, Werngren J, Machado D, Borroni E, Wijkander M, Lina G, Mouton J, Matuschek E, Kahlmeter G, Giske C, Santin M, Cirillo DM, Viveiros M, Cambau E. 2020. Antimicrobial susceptibility testing of Mycobacterium tuberculosis complex isolates - the EUCAST broth microdilution reference method for MIC determination. Clin Microbiol Infect 26:1488–1492. doi:10.1016/j.cmi.2020.07.036 32750539

[11] European Committee on Antimicrobial Susceptibility Testing. 2019. MIC distributions and epidemiological cut-off value (ECOFF) setting, EUCAST SOP 10.1. http://www.eucast.org.

[12] Kahlmeter G. 2015. The 2014 Garrod Lecture: EUCAST - are we heading towards international agreement? J Antimicrob Chemother 70:2427–2439. doi:10.1093/jac/dkv145 26089441

[13] CRyPTIC Consortium. 2022. Epidemiological cut-off values for a 96-well broth microdilution plate for high-throughput research antibiotic susceptibility testing of M. tuberculosis. Eur Respir J 60:2200239. doi:10.1183/13993003.00239-2022 35301246 PMC9556810

[14] Kahlmeter G, Turnidge J. 2023. The determination of epidemiological cut-off values requires a systematic and joint approach based on quality controlled, non-truncated minimum inhibitory concentration series. Eur Respir J 61:2202259. doi:10.1183/13993003.02259-2022 37147008

[15] Köser CU, Maurer FP. 2023. Minimum inhibitory concentrations and sequencing data have to be analysed in more detail to set provisional epidemiological cut-off values for Mycobacterium tuberculosis complex. Eur Respir J 61:2202397. doi:10.1183/13993003.02397-2022 37147011

[16] CRyPTIC Consortium. 2023. Reply: epidemiological cut-off values for a 96-well broth microdilution plate for high-throughput research antibiotic susceptibility testing of M. tuberculosis. Eur Respir J 61:2300426. doi:10.1183/13993003.00426-2023 37147010 PMC10160797

[17] WHO. 2018. Technical report on critical concentrations for drug susceptibility testing of medicines used in the treatment of drug-resistant tuberculosis.

[18] Feuerriegel S, Schleusener V, Beckert P, Kohl TA, Miotto P, Cirillo DM, Cabibbe AM, Niemann S, Fellenberg K. 2015. PhyResSE: a web tool delineating Mycobacterium tuberculosis antibiotic resistance and lineage from whole-genome sequencing data. J Clin Microbiol 53:1908–1914. doi:10.1128/JCM.00025-15 25854485 PMC4432036

[19] Phelan JE, O’Sullivan DM, Machado D, Ramos J, Oppong YEA, Campino S, O’Grady J, McNerney R, Hibberd ML, Viveiros M, Huggett JF, Clark TG. 2019. Integrating informatics tools and portable sequencing technology for rapid detection of resistance to anti-tuberculous drugs. Genome Med 11:41. doi:10.1186/s13073-019-0650-x 31234910 PMC6591855

[20] Pankhurst LJ, Del Ojo Elias C, Votintseva AA, Walker TM, Cole K, Davies J, Fermont JM, Gascoyne-Binzi DM, Kohl TA, Kong C, Lemaitre N, Niemann S, Paul J, Rogers TR, Roycroft E, Smith EG, Supply P, Tang P, Wilcox MH, Wordsworth S, Wyllie D, Xu L, Crook DW, COMPASS-TB Study Group. 2016. Rapid, comprehensive, and affordable mycobacterial diagnosis with whole-genome sequencing: a prospective study. Lancet Respir Med 4:49–58. doi:10.1016/S2213-2600(15)00466-X 26669893 PMC4698465

[21] Jajou R, van der Laan T, de Zwaan R, Kamst M, Mulder A, de Neeling A, Anthony R, van Soolingen D. 2019. WGS more accurately predicts susceptibility of Mycobacterium tuberculosis to first-line drugs than phenotypic testing. J Antimicrob Chemother 74:2605–2616. doi:10.1093/jac/dkz215 31119271

[22] Shea J, Halse TA, Modestil H, Kearns C, Fowler RC, Da Costa-Carter C-A, Siemetzki-Kapoor U, Leisner M, Lapierre P, Kohlerschmidt D, Rowlinson M-C, Escuyer V, Musser KA. 2023. Mycobacterium tuberculosis complex whole-genome sequencing in New York State: implementation of a reduced phenotypic drug susceptibility testing algorithm. Tuberculosis (Edinb) 142:102380. doi:10.1016/j.tube.2023.102380 37543009

[23] Anthony R, Groenheit R, Mansjö M, de Zwaan R, Werngren J. 2023. The relative positioning of genotyping and phenotyping for tuberculosis resistance screening in two EU national reference laboratories in 2023. Microorganisms 11:1809. doi:10.3390/microorganisms11071809 37512981 PMC10383358

[24] Folkhälsomyndigheten. Tuberculosis - disease statistics. Folkhälsomyndigheten. Available from: https://www.folkhalsomyndigheten.se/folkhalsorapportering-statistik/statistik-a-o/sjukdomsstatistik/tuberkulos/?year%5B%5D=&scope%5B%5D=all&tab=tab-region

[25] Coll F, McNerney R, Guerra-Assunção JA, Glynn JR, Perdigão J, Viveiros M, Portugal I, Pain A, Martin N, Clark TG. 2014. A robust SNP barcode for typing Mycobacterium tuberculosis complex strains. Nat Commun 5:4812. doi:10.1038/ncomms5812 25176035 PMC4166679

[26] Mansjö M, Karlsson Lindsjö O, Grönfors Seeth C, Groenheit R, Werngren J. 2022. The ddn Trp20Stop mutation and its association with lineage 4.5 and resistance to delamanid and pretomanid in Mycobacterium tuberculosis. Antimicrob Agents Chemother 66:e0102622. doi:10.1128/aac.01026-22 36409105 PMC9765023

[27] WHO. Catalogue of mutations in Mycobacterium tuberculosis complex and their association with drug resistance

[28] R Core Team. 2013. R: a language and environment for statistical computing. Foundation for statistical computing. Vienna, Austria. https://www.R-project.org.

[29] Wickham H, Averick M, Bryan J, Chang W, McGowan L, François R, Grolemund G, Hayes A, Henry L, Hester J, Kuhn M, Pedersen T, Miller E, Bache S, Müller K, Ooms J, Robinson D, Seidel D, Spinu V, Takahashi K, Vaughan D, Wilke C, Woo K, Yutani H. 2019. Welcome to the Tidyverse. J Open Source Software 4:1686. doi:10.21105/joss.01686

[30] Stevenson M, Sergeant E, Firestone S. 2023. epiR: tools for the analysis of epidemiological data. https://CRAN.R-project.org/package=epiR.

[31] Chen JM, Uplekar S, Gordon SV, Cole ST. 2012. A point mutation in cycA partially contributes to the D-cycloserine resistance trait of Mycobacterium bovis BCG vaccine strains. PLoS One 7:e43467. doi:10.1371/journal.pone.0043467 22912881 PMC3422274

[32] Durek C, Rüsch-Gerdes S, Jocham D, Böhle A. 2000. Sensitivity of BCG to modern antibiotics. Eur Urol 37:21–25. doi:10.1159/000052378 10575268

[33] World Health Organization. ISBN: 9789240090491. WHO operational handbook on tuberculosis: module 3: diagnosis: rapid diagnostics for tuberculosis detection: web annex B: critical concentrations for pretomanid and cycloserine: WHO policy statement. 3rd ed

[34] WHO. 2023. ISBN: 9789240082410. Catalogue of mutations in Mycobacterium tuberculosis complex and their association with drug resistance. 2nd ed

[35] Ritz N, Tebruegge M, Connell TG, Sievers A, Robins-Browne R, Curtis N. 2009. Susceptibility of Mycobacterium bovis BCG vaccine strains to antituberculous antibiotics. Antimicrob Agents Chemother 53:316–318. doi:10.1128/AAC.01302-08 18955515 PMC2612166

[36] European Committee on Antimicrobial Susceptibility Testing. 2021. EUCAST reading guide for broth microdilution. Version 3.0. http://www.eucast.org.

[37] WHO. 2022. ISBN: 978-92-4-004741-9. Optimized broth microdilution plate methodology for drug susceptibility testing of Mycobacterium tuberculosis complex

[38] Sassetti CM, Boyd DH, Rubin EJ. 2003. Genes required for mycobacterial growth defined by high density mutagenesis. Mol Microbiol 48:77–84. doi:10.1046/j.1365-2958.2003.03425.x 12657046

[39] European Committee on Antimicrobial Susceptibility Testing. 2019. Area of Technical Uncertainty (ATU) in antimicrobial susceptibility testing

[40] Clinical and Laboratory Standards Institute. 2018. CLSI supplement M62. Performance standards for susceptibility testing of mycobacteria, Nocardia spp., and other aerobic actinomycetes. 1st ed

[41] Bateson A, Ortiz Canseco J, McHugh TD, Witney AA, Feuerriegel S, Merker M, Kohl TA, Utpatel C, Niemann S, Andres S, et al.. 2022. Ancient and recent differences in the intrinsic susceptibility of Mycobacterium tuberculosis complex to pretomanid. J Antimicrob Chemother 77:1685–1693. doi:10.1093/jac/dkac070 35260883 PMC9155602

[42] Rupasinghe P, Reenaers R, Vereecken J, Mulders W, Cogneau S, Merker M, Niemann S, Vally Omar S, Rigouts L, Köser CU, Decroo T, de Jong BC. 2024. Refined understanding of the impact of the Mycobacterium tuberculosis complex diversity on the intrinsic susceptibility to pretomanid. Microbiol Spectr 12:e0007024. doi:10.1128/spectrum.00070-24 38334384 PMC10913522

[43] European Committee on Antimicrobial Susceptibility Testing. 2021. MIC distributions and epidemiological cut-off value (ECOFF) setting, EUCAST SOP 10.2. Available from: http://www.eucast.org

