# Supplementary material for: Performance of a broth microdilution assay for routine minimum inhibitory concentration determination of 14 anti-tuberculous drugs against the Mycobacterium tuberculosis complex based on the EUCAST reference protocol
Source: Antimicrob Agents Chemother. 2024 Dec 18;69(2):e00946-24. doi: 10.1128/aac.00946-24 (PMC11823602; doi:10.1128/aac.00946-24)
Supplement: Supplemental methods — Detailed description of the BMD protocol. [file aac.00946-24-s0001.docx]

**Supplementary method: Broth Microdilution (BMD) protocol for antimicrobial susceptibility testing of *Mycobacterium tuberculosis* complex strains**

Preparation for MIC test of water-soluble agents

Stock solutions of isoniazid (INH), rifampicin (RIF), ethambutol (EMB), amikacin (AMI), moxifloxacin (MFX), levofloxacin (LFX), para-aminosalicylic acid (PAS), linezolid (LZD), ethionamide (ETH) and cycloserine (CS) were prepared at 40X the final test concentration and stored as 1 mL aliquots at -70°C (details on the preparation of the stock solutions are presented in Supplementary table S4).

At the day of MIC determination, 100 µL of Middlebrook 7H9 with 10% OADC (oleic acid dextrose catalase) and 0.2% glycerol (7H9/OADC) was added to the growth controls (GC1% and GC 100%) and antibiotic-containing wells in a round bottom untreated polystyrene microplate (Falcon® 96-well Clear Round Bottom Not Treated Microplate, with Lid, art nr 351177, Corning, New York, USA). Two hundred microliter of 7H9/OADC was added to all negative controls and 200 µL of sterile water was added to the remaining peripheral wells (Figure 1).

The stock solutions of the water-soluble antibiotics were thawed to reach room temperature and then diluted in 7H9/OADC to 4X the final test concentration (working dilutions). One hundred microliter of each drug working dilution was added to the row (2B-G) in the microtiter plate which corresponds to the highest test concentration. Thereafter nine 1:2 dilutions were made by adding 100 µL of the antibiotic solution present in the highest concentration row to the following row/wells (3B-G), applying the same procedure throughout the next rows/wells and finally discard the last 100 µL of the last row/wells (11B-G).

Preparation for MIC test of water-insoluble agents

Ten stock solutions at 200X the final test concentrations were made for bedaquiline (BDQ), clofazimine (CFZ), delamanid (DLM) and pretomanid (PA) in 100% DMSO (dimethyl sulfoxide) and stored as 0.2 mL aliquots in untreated polystyrene 96-well plates (Falcon® 96-well Clear Round Bottom Not Treated Microplate, with Lid, art nr 351177, Corning, New York, USA) at -70°C (details on the preparation of the stock solutions are presented in Supplementary table S4).

At the day of MIC determination 100 µL of 7H9/OADC with 1% DMSO was added to both growth controls (GC1% and GC100%), 200 µL of 7H9/OADC with 1% DMSO was added to all negative controls and 200 µL of sterile water was added to the remaining peripheral wells (Figure 1).

The stock solutions of the water-insoluble antibiotics were thawed to reach room temperature and then diluted in 7H9/OADC to 2X the final test concentration (working dilutions) in glass vials. One hundred microliter of each drug working dilution was added to the corresponding antibiotic-containing well in the microtiter plate as presented in the plate layout (Plate 3) in Figure 1.

Inoculation of the microtiter plate

Bacterial inoculates were prepared from cultures growing on Löwenstein-Jensen medium within two weeks after visible growth. Briefly, approximately 5 µL of bacteria were vortexed in a glass tube containing 5-10 glass beads for two minutes before resuspension in 5 mL of sterile water. The bacterial suspension was vortexed for another two minutes and left to sediment for 30 minutes. The supernatant was adjusted to a turbidity of 0.5 McFarland (McF) (by using a nephelometer) in a new glass tube and then diluted 10^-1^, 10^-2^, 10^-3^ and 10^-4^ in Middlebrook 7H9/OADC as described in the EUCAST reference protocol (1).

One hundred microliter of the 10^-2^-diluted 0.5 McF bacterial suspensions was added to the antibiotic-containing wells and the GC100% and 100 µL of the 10^-4^-diluted 0.5 McF bacterial suspensions was added to the GC1%.

The plates were incubated at 36°C ± 1°C and read at day 7, 10, 14 and 21. The MICs were determined at the point the GC1% well showed visible growth.

The reference strain H37Rv ATCC 27294 was tested at eight different occasions to examine the technical reproducibility of the assay. As an additional means of assessing the inoculum density, the colony forming unit (CFU) counts were determined on Middlebrook 7H10 agar for the H37Rv replicates.

**References**

1. Schön T, Werngren J, Machado D, Borroni E, Wijkander M, Lina G, Mouton J, Matuschek E, Kahlmeter G, Giske C, Santin M, Cirillo DM, Viveiros M, Cambau E. 2020. Antimicrobial susceptibility testing of *Mycobacterium tuberculosis* complex isolates - the EUCAST broth microdilution reference method for MIC determination. Clin Microbiol Infect 26:1488-1492.
